# Fresh (Non-cryopreserved) autologous stem cell transplantation in multiple myeloma: Faster engraftment and reduced hospitalization

**DOI:** 10.1016/j.htct.2026.106513

**Published:** 2026-09-08

**Authors:** Merve Kakci, Osman Can Öztürk, Ömer Şeker, Semih Basci, Hikmetullah Batgi, Sinem Namdaroğlu

**Affiliations:** Department of Hematology, Dokuz Eylül University Faculty of Medicine, İzmir, Türkiye

**Keywords:** Multiple myeloma, Autologous stem cell transplantation, Fresh peripheral stem cells, DMSO-free, Engraftment, Cost analysis

## Abstract

**Background:**

Cryopreserved autologous hematopoietic stem cell transplantation using dimethyl sulfoxide is standard in multiple myeloma but it is associated with infusion-related toxicity and high cost. This study aimed to evaluate the feasibility, safety, and outcomes of dimethyl sulfoxide-free, fresh (non-cryopreserved) autologous stem cell transplantation in multiple myeloma patients at a single tertiary center.

**Methods:**

A retrospective analysis was conducted in 40 multiple myeloma patients who underwent transplantation using freshly collected, non-cryopreserved peripheral blood stem cells between 2023 and 2025 at Dokuz Eylül University Hospital. Peripheral blood stem cells were infused within 72 h after collection without cryopreservation. Engraftment kinetics, toxicity, and length of hospital stay were assessed descriptively.

**Results:**

All patients achieved hematopoietic engraftment (100%). Median neutrophil and platelet engraftment occurred at nine (range: 6–14) and 11 days (range: 7–16), respectively and the median hospitalization was 13 days. No infusion-related adverse events or grade 3–4 mucositis were observed. This approach eliminated dimethyl sulfoxide-related toxicity, reduced cryopreservation-related resource utilization and associated procedural costs.

**Conclusion:**

Fresh, dimethyl sulfoxide-free autologous stem cell transplantation in multiple myeloma is a safe, effective, and cost-efficient alternative to cryopreserved transplantation. It provides rapid engraftment, reduced hospitalization, and improved patient comfort, representing a practical model for modern transplant centers.

## Introduction

Multiple myeloma (MM) has evolved from a rapidly fatal malignancy into a biologically complex and increasingly manageable disease driven by continuous therapeutic innovation. The integration of proteasome inhibitors, immunomodulatory agents, monoclonal antibodies, and emerging cellular therapies has markedly improved patient outcomes; nevertheless, high-dose melphalan followed by autologous hematopoietic stem cell transplantation (ASCT) remains a central pillar of therapy for transplant-eligible patients, consistently deepening responses and extending progression-free survival [[1], [2], [3]]. While systemic treatment strategies have advanced substantially, many technical aspects of the transplantation workflow itself have changed little over decades, prompting renewed scrutiny of whether established logistical conventions still reflect optimal contemporary practice.

Routine cryopreservation of peripheral blood stem cells (PBSCs) using dimethyl sulfoxide (DMSO) represents one of the most deeply embedded components of standard ASCT. Historically introduced to provide scheduling flexibility and ensure graft availability, cryopreservation has become widely accepted despite accumulating evidence suggesting potential biological and clinical disadvantages. DMSO exposure is associated with a well-recognized spectrum of infusion-related toxicities, including nausea, vomiting, cardiovascular instability, and organ-specific adverse effects, that directly impact patient experience during transplantation [4,5]. Moreover, experimental and translational data indicate that freeze-thaw stress may induce cellular injury, apoptosis, and immune dysregulation, raising the possibility that cryopreservation is not entirely biologically neutral [6,7]. In parallel, the cryogenic process imposes significant logistical and economic demands, requiring specialized infrastructure, liquid nitrogen storage, laboratory processing, and additional consumables [8].

Advances in mobilization strategies, conditioning regimens, and transplant coordination have reintroduced interest in fresh, non-cryopreserved PBSC infusion. By eliminating DMSO exposure and avoiding freeze-thaw injury, fresh transplantation offers a streamlined workflow with the potential to improve tolerability without compromising efficacy. Contemporary studies suggest that fresh grafts achieve reliable and, in selected cohorts, accelerated hematopoietic recovery, accompanied by reduced gastrointestinal toxicity, lower mucositis burden, and fewer infusion-related adverse events [[9], [10], [11]]. These observations challenge the long-standing perception of cryopreservation as an obligatory safeguard and instead position it as a logistical solution that may not be universally required.

The implications of this evolving perspective extend beyond toxicity profiles alone. As transplant programs face increasing pressure to balance clinical efficacy with cost sustainability and operational efficiency, approaches that simultaneously enhance patient comfort, reduce toxicity, and simplify infrastructure warrant critical evaluation. DMSO-free fresh ASCT aligns with emerging priorities in modern hematology, including precision-driven care, procedural efficiency, and resource optimization [[12], [13], [14], [15]]. Despite growing international experience, comprehensive real-world analyses focusing on early engraftment kinetics, toxicity burden, and hospitalization outcomes remain limited, and data from Türkiye are particularly scarce.

Forty consecutive MM patients undergoing ASCT using freshly collected, DMSO-free PBSCs were evaluated in this retrospective single-center study. We hypothesized that elimination of cryopreservation would result in earlier engraftment, reduced nausea, vomiting, and mucositis, absence of DMSO-related toxicities, and reduced length of hospital stay. Rather than addressing feasibility alone, this study aims to explore whether a simplified transplantation workflow can deliver measurable clinical advantages while maintaining transplant safety.

Taken together, these considerations prompt a broader question for contemporary transplant practice: in an era prioritizing precision, tolerability, and sustainability, should routine cryopreservation remain the default strategy, or is fresh, DMSO-free transplantation poised to redefine the standard ASCT paradigm in carefully selected patients?

## Materials and methods

### Study design and patient population

This retrospective single-center observational study was conducted at the Bone Marrow Transplantation Unit of Dokuz Eylül University, a tertiary referral center in İzmir, Türkiye. Adult patients (≥18 years) with a confirmed diagnosis of MM who underwent ASCT using freshly collected, non-cryopreserved (DMSO-free) PBSC between 2023 and 2025 were consecutively included. Transplant eligibility and response assessment were based on International Myeloma Working Group (IMWG) criteria, and only patients with at least stable disease prior to transplantation were eligible for inclusion [16].

### Stem cell mobilization and collection

Mobilization was performed using granulocyte colony-stimulating factor (G-CSF) at 10 µg/kg/day administered subcutaneously for five consecutive days, either alone or combined with plerixafor (0.24 mg/kg) in patients with predicted poor mobilization according to institutional practice [17]. Peripheral blood CD34⁺ counts were monitored daily starting on Day 4, and leukapheresis was initiated when circulating CD34⁺ cells exceeded 20 × 10⁶ cells/L using continuous-flow apheresis systems. The minimum target yield was ≥2 × 10⁶ CD34⁺ cells/kg. Collected grafts were stored at 4 °C in sterile transfusion bags and infused within 72 h without cryopreservation or exposure to DMSO in accordance with previously described fresh PBSC transplantation protocols [18].

### Conditioning regimen and transplant procedure

High-dose melphalan was administered as the conditioning regimen at 200 mg/m² on Day −1. In patients with renal impairment (creatinine clearance <50 mL/min), the dose was reduced to 140 mg/m² according to established transplant practice guidelines [19]. PBSC, without cryopreservation or DMSO exposure, were infused on Day 0 via a central venous catheter over 15–30 min under continuous vital sign monitoring.

### Supportive care

All patients received institutional standard prophylaxis, including antibacterial (levofloxacin), antifungal (fluconazole), and antiviral (valaciclovir) therapy consistent with international supportive care recommendations for ASCT recipients [20]. G-CSF at 5 µg/kg/day was initiated when leukocyte counts declined below 1.0 × 10⁹/L and continued until neutrophil engraftment. Patients were monitored daily for infectious complications, mucositis, gastrointestinal toxicity, and infusion-related adverse events.

### Definitions and outcomes

Neutrophil engraftment was defined as the first of three consecutive days with an absolute neutrophil count (ANC) ≥0.5 × 10⁹/L, and platelet engraftment as the first of three consecutive days with platelet counts ≥20 × 10⁹/L without transfusion support [21]. Adverse events, including mucositis, nausea, vomiting, and infusion-related reactions, were graded using the National Cancer Institute Common Terminology Criteria for Adverse Events (CTCAE), version 5.0 [22]. Length of hospitalization was defined as the interval from transplant admission to discharge following hematopoietic recovery and resolution of major complications.

### Data collection

Demographic, clinical, and laboratory variables were retrospectively extracted from institutional electronic health records and the bone marrow transplantation registry. Collected variables included age, sex, disease stage, induction regimen, mobilization strategy, CD34⁺ cell yield, engraftment kinetics, toxicity incidence, and length of hospital stay.

### Statistical analysis

Descriptive statistics were used to summarize baseline characteristics and outcomes. Categorical variables are expressed as frequencies and percentages, whereas continuous variables are summarized as medians with ranges. Given the limited follow-up and absence of relapse or mortality events during the observation period, time-to-event analyses such as overall survival or event-free survival could not be performed. The primary objective was to evaluate procedural safety, tolerability, engraftment kinetics, toxicity profile, and length of hospital stay associated with fresh, DMSO-free ASCT rather than long-term therapeutic efficacy.

## Results

### Patient characteristics

Patient characteristics are summarized in Table 1. A total of 40 consecutive MM patients underwent ASCT using freshly collected, non‑cryopreserved PBSC. Induction therapy consisted primarily of VCD (velcade, cyclophosphamide, dexamethasone) in 27 patients (67.5%) and VRD (velcade, revlimid, dexamethasone) in 11 patients (27.5%), while two patients received alternative regimens. Prior radiotherapy exposure was rare (2.5%). Stem cell mobilization was successful in all cases, including patients previously treated with lenalidomide-based induction. Notably, none of the patients had received anti‑CD38 monoclonal antibodies therapy prior to mobilization.

**Table 1:** Baseline Patient Characteristics (n = 40).

Variable	
Age (years) - median (range)	64 (43–72)
Gender, M/F - n (%)	21 (52.5) / 19 (47.5)
BMI (kg/m²) - median (range)	26 (21–40)
ECOG performance status - median (range)	0 (0–1)
Primary diagnosis – Multiple myeloma	40 (100)
Pre-transplant chemotherapy line - median (range)	1 (1–2)
VCD regimen - n (%)	27 (67.5)
VRD regimen - n (%)	11 (27.5)
Other regimens - n (%)	2 (5.0)
Radiotherapy received - n (%)	1 (2.5)
Disease status before transplant - n (%)	
Complete response	12 (30.0)
Very good partial response	15 (37.5)
Partial response	12 (30.0)
Stable disease	1 (2.5)
Revised ISS stage - n (%)	
Stage I	10 (25.0)
Stage II	18 (45.0)
Stage III	12 (30.0)
Myeloma subtype - n	
Heavy chain (IgG/IgA/IgM)	26 / 6 / 1
Light chain (κ/λ)	5 / 2

ECOG: Eastern Cooperative Oncology Group; BMI: Body Mass Index; CR: complete response; VGPR: very good partial response; PR: partial response; R-ISS: Revised International Staging System; Ig: immunoglobulin; VCD: bortezomib–cyclophosphamide–dexamethasone; VRD: bortezomib–lenalidomide–dexamethasone.

At the time of transplantation, disease responses included complete response in 12 patients (30%), very good partial response in 15 (37.5%), partial response in 12 (30%), and stable disease in one patient (2.5%). According to the Revised International Staging System, 25% of patients were Stage I, 45% in Stage II, and 30% in Stage III. Heavy‑chain myeloma predominated (IgG: n = 26, IgA: n = 6, IgM: n = 1), whereas seven patients presented with light‑chain disease.

### Transplant characteristics

Key transplant-related variables are summarized in Table 2. The median Hematopoietic Cell Transplantation Comorbidity Index was 0 (range: 0–2), and the median Revised International Staging System Stage was II. Mobilization was performed using G‑CSF alone in 26 patients (65%) and G‑CSF plus plerixafor in 14 patients (35%). All patients received high‑dose melphalan conditioning. The median infused CD34⁺ cell dose was 6.3 × 10⁶/kg (range: 3.1–11), thereby exceeding standard minimum thresholds for autologous transplantation. Baseline hematologic parameters prior to conditioning were within clinically acceptable limits across the cohort.

**Table 2:** Transplant and Laboratory Characteristics (n = 40).

**Variable**	
HCT-CI score - median (range)	0 (0–2)
age-adjusted HCT-CI score	1 (1–3)
Mobilization regimen	
G-CSF alone - n (%)	26 (65.0)
G-CSF plus plerixafor - n (%)	14 (35.0)
Conditioning regimen (melphalan) - n (%)	40 (100)
Infused CD34^+^ (cells × 10⁶/kg) - median (range)	6.3 (3.1–11.0)
Hemoglobin (g/dL) - median (range)	12.2 (8.9–14.3)
WBC (×10⁹/L) - median (range)	6.1 (2.7–12.1)
Neutrophil (×10⁹/L) - median (range)	4.1 (1.2–10.5)
Lymphocyte (×10⁹/L) - median (range)	1.2 (0.3–5.8)
Platelet (×10⁹/L) - median (range)	302 (124–462)
LDH (U/L) - median (range)	183 (133–308)
Ferritin, ng/mL - median (range)	83 (14–5700)
Engraftment achieved - n (%)	40 (100)
Neutrophil engraftment (days) - median (range)	9 (6–14)
Platelet engraftment (days) - median (range)	11 (7–16)
Hospitalization (days) - median (range)	13 (9–18)
Anti-CD38 exposure pre-mobilization	None
Diagnosis to ASCT (months) - median (range)	5 (4–7)
RBC transfusions (units) - median (range)	0 (0–3)
Platelet transfusions (units) - median (range)	3 (2–7)
Systemic antibiotic episodes (per patient) - median (range)	1 (0–2)

HCT-CI: Hematopoietic Cell Transplantation Comorbidity Index; CD34: cluster of differentiation 34; LDH: lactate dehydrogenase; WBC: white blood cells; G-CSF: granulocyte colony-stimulating factor.

### Hematopoietic engraftment

Successful hematopoietic engraftment was achieved in all patients (100%). Median neutrophil recovery occurred at a median of Day 9 (range: 6–14), while platelet engraftment was observed at a median of Day 11 (range: 7–16). No cases of graft failure, delayed engraftment, or engraftment syndrome were documented. Overall engraftment kinetics appeared numerically favorable compared with historical benchmarks reported in cryopreserved ASCT series.

### Safety and toxicity

The DMSO‑free transplantation approach demonstrated an excellent safety profile. No infusion‑related adverse events (nausea, vomiting, bradycardia, hypotension, or acute infusion reactions) were observed during stem cell administration. Early transplant-related toxicity was limited, and no patients required intensive care unit admission. Importantly, there were no early transplant‑related deaths within the first 100 days. The median length of hospital stay was 13 days (range: 9–18), reflecting efficient hematopoietic recovery and favorable clinical tolerability.

### Additional resource-utilization outcomes

The median time from diagnosis to ASCT was five months (range: 4–7). Patients required a median of zero red blood cell units (range: 0–3) and three platelet units (range: 2–7). Additionally, systemic antibiotics were administered to 27 patients (67.5%), with a median of one episode per patient (range: 0–2).

### Operational and cost‑related observations

The use of freshly collected PBSCs eliminated the need for cryopreservation, including freezing consumables, liquid nitrogen storage, and a controlled‑rate freezing infrastructure. This simplified the transplantation workflow and reduced preparation time within the transplant unit. Operationally, fresh ASCT was associated with improved logistical efficiency while maintaining consistent clinical outcomes, supporting its potential as a streamlined and resource‑efficient transplantation strategy.

## Discussion

ASCT remains a cornerstone of therapy for transplant-eligible MM patients [[1], [2], [3]]. However, as therapeutic paradigms evolve toward improved tolerability and patient-centered care, increasing attention has been directed toward optimizing the procedural aspects of transplantation itself. In this single-center real-world cohort, ASCT performed with freshly collected, non-cryopreserved PBSC demonstrated rapid and consistent engraftment, absence of infusion-related toxicity, and a reduced length of hospital stay. Collectively, these findings support the feasibility of a coordinated fresh transplantation strategy and suggest that elimination of DMSO exposure may confer clinically meaningful advantages without compromising transplant reliability.

### Comparison with conventional cryopreserved hematopoietic stem cell transplantation

Conventional cryopreserved ASCT series typically report median neutrophil engraftment at approximately 10–12 days and platelet engraftment at 12–16 days, with inpatient stays frequently ranging from 15–20 days [14,23,24]. Moreover, infusion of DMSO-containing grafts is associated with adverse events, including nausea, vomiting, hypotension, bradycardia, and occasional cardiopulmonary complications, with infusion-related toxicity rates reaching 20%−40% in some cohorts [25,26]. In contrast, the current cohort demonstrated median neutrophil recovery at nine days, platelet engraftment at 11 days, and a median hospitalization of 13 days, suggesting numerically earlier hematopoietic recovery and streamlined post-transplant care.

### Contextualization within global fresh transplant experience

Interest in fresh, non-cryopreserved ASCT has re-emerged internationally as advances in mobilization strategies and transplant coordination have enabled reliable same-cycle graft infusion [[27], [28], [29]]. Prior observational studies have reported comparable, or in some cases accelerated, engraftment kinetics with fresh transplantations, alongside reduced infusion-related adverse events and improved patient comfort. The findings of this study are consistent with these reports.

### National experience and relevance

Data on DMSO-free ASCT from Türkiye remain limited, and most centers continue to rely on cryopreservation as standard practice [15,30]. The present cohort represents one of the most homogeneous MM populations reported nationally using a fresh transplantation strategy.

### Toxicity, quality of life, and the emergence of outpatient-oriented transplant models

As MM increasingly resembles a chronic disease, treatment decisions are shaped not only by efficacy but also by patient experience and quality of life [2,3]. Eliminating DMSO exposure removes a major contributor to infusion-related discomfort, particularly nausea and vomiting. The rapid engraftment and shortened hospitalization observed in the present cohort suggest that, under appropriate institutional conditions, fresh ASCT may facilitate early discharge or even outpatient-based transplant models.

### Operational and health-economic considerations

Cryopreservation requires controlled-rate freezing systems, liquid nitrogen storage, cryogenic consumables, and additional laboratory handling, all of which increase procedural complexity and cost [12]. Previous reports have suggested that fresh ASCT may reduce transplantation expenses through elimination of these requirements, with estimated cost reductions of up to 25%−30% in some analyses [31].

### Study limitations

Several limitations should be acknowledged. The retrospective, single-center design and relatively small sample size may limit generalizability, and the absence of a contemporaneous cryopreserved control group precludes definitive comparative conclusions. Long-term outcomes, including progression-free and overall survival, were not assessed.

### Clinical implications and future directions

Taken together, these results contribute to a growing body of evidence suggesting that fresh, DMSO-free ASCT may represent a safe, effective, and potentially resource-efficient alternative to conventional cryopreserved transplantation. Future studies incorporating prospective comparisons, patient-reported outcomes, and health-economic analyses are essential.

## Conclusion

In summary, freshly collected, DMSO-free ASCT in MM is associated with rapid engraftment, excellent tolerability, and reduced length of hospital stay in this real-world cohort, supporting the concept that ASCT can increasingly function as a low-toxicity therapeutic step within modern myeloma treatment.

## Funding

None

## Ethical approval

Informed consent was not deemed necessary due to the retrospective nature of the study and the use of anonymized data.

## Data availability

The data that support the findings of this study are available from the corresponding author upon reasonable request.

## Conflicts of interest

None declared.
